# Delivery without Rupture Hymen

**Published:** 1874-02

**Authors:** R. Beverly Cole

**Affiliations:** Professor of Obstetrics and Clinical Diseases of Women, University of California


*THE WESTERN LANCET—December*, 1873:

Pregnancy and Delivery Without Rupture of the Hymen.—*By R. Beverly Cole, Professor of Obstetrics and Clinical Diseases of Women, University of California.*—On examination, after inspecting the abdominal tumor, we encountered at or within the vulva, the hymen unruptured, and presenting a thick, semi-cartilaginous margin, with an orifice barely sufficient to admit of the passage of the finger—indeed, some difficulty was experienced in forcing the finger through it, and which occasioned considerable pain.

We therefore charged her with having told us an untruth, but she persisted in saying that, although her husband had *attempted* to cohabit with her, yet he had entirely failed in entering the vagina, and for this reason she had never suspected herself to be *enciente*.

Three hours later we called again; the pains were now such as characterize the second stage of labor, and we found, on examination, the head had engaged at the superior strait and was descending rapidly.

The next pain pushed forward the tumor and vulva, but made no impression upon the hymen. We felt it was of the first importance to support the perinæum, and direct the advancing head in accordance with the "curve of carus." Each pain was now more severe and they were becoming double, yet no dilatation of the vulva was perceptible, and whilst we were admonishing the patient against using any exertion (fearing a laceration of the perinæum) another most violent pain was inaugurated, and the head, in spite of every effort of ours, was forced through the perinæum, carrying with it a loop of the funis, and without lacerating in the slightest degree the posterior commissure of the vulva anteriorly, or the sphincter ani posteriorly; immediately followed the shoulders and body, and after separating the child in the usual manner, we concluded to deliver as is our practice in all cases, the placenta. The hand was introduced through the laceration in the perinæum, carried to the cavity of the uterus, the placenta seized and delivered through the accidental opening.

The after conduct of the case was the same as is usual under ordinary circumstances. The lacerated opening in the perinæum contracted so that in three hours after, when we examined her, it was not larger than would be necessary to admit the finger, and at the usual time of getting up it had diminished until it was no larger than a goose quill, and is now not nearly so large. We have repeatedly urged her to have the slight fistulous opening closed by operative procedure, but she has always declined. No subsequent pregnancy has occurred, and she is an example of a woman who is a mother and yet retains all the (at one time considered) necessary evidences of virginity.

